# Diffusion MRI as A Biomarker for Monitoring Recovery After Surgical Repair of Traumatic Peripheral Nerve Injuries: A Longitudinal Case Series

**DOI:** 10.21203/rs.3.rs-9282445/v1

**Published:** 2026-04-28

**Authors:** Isaac V. Manzanera Esteve, Barite Gutama, Ronald M. Cornely, Yan Ling, Kezia Sharon Christopher, Ping Wang, Kristianna Lombardi, Galen Perdikis, Mark A. Mahan, Richard Dortch, Wesley P. Thayer

**Affiliations:** 1 Department of Plastic Surgery, Vanderbilt Health, Nashville, Tennessee, USA; 2 Department of Neuroimaging Innovation Center, Barrow Neurological Institute, Phoenix, Arizona, USA; 3 Department of Neurosurgery, Clinical Neurosciences Center, University of Utah, Salt Lake City, UT, USA.

## Abstract

After transection, peripheral nerve regeneration requires repair surgery in a timely manner. Unfortunately, up to 40% of peripheral nerve repairs fail to achieve functional recovery. Patients with failed peripheral nerve repairs require additional surgical interventions, but current methods detecting clinical failure offer limited information, resulting in delayed management and suboptimal outcomes. Diffusion tensor imaging can serve as a biomarker of nerve regeneration; however, the complex spatial and temporal changes along the distal segment of the nerve remains challenging to interpret. To address this, we applied the Gompertz function to automatically characterize these changes and capture the nonlinear dynamics of recovery following nerve trauma.

Severe peripheral nerve injuries of the ulnar or median nerve were analyzed in this study. Medical Research Council sensory grades representing absent to normal sensation, motor grades representing visible muscle contraction to normal strength and diffusion tensor imaging scans were performed longitudinally over twelve months postoperatively. Values of fractional anisotropy closely matched clinical evaluations and levels of nerve recovery.

We provided an automated, noninvasive approach for characterizing recovery and reducing user-dependent variability in interpretation. These results support Fractional Anisotropy as a potential biomarker to monitor nerve recovery after surgical repair, potentially guiding clinical decisions for additional interventions.

## Introduction:

Traumatic peripheral nerve injuries (TPNIs) have an incidence of 16.9 per 100,000 people in the United States per year, accounting for 1% to 3% of trauma patients^1^. TPNI recovery can take months, with associated sensory and motor sequelae that result in chronic pain disability and hinder quality of life^2^. Unfortunately, in up to 40% of severe peripheral nerve injuries, surgical repairs fail to achieve functional recovery, and a secondary repair is required^3^. Additionally, there is a 1% loss of achievable sensory and motor function every 6 days of delay in diagnosis and surgical intervention^4^. Currently, there is a lack of reliable non-invasive diagnostic tools to monitor postoperative nerve recovery and identify, at an early stage, patients with failed regeneration who may benefit from additional surgical intervention^3^.

Recovery following peripheral nerve injury is a slow and complex process. In cases of neurotmesis, where the nerve trunk is severed, the axons undergo Wallerian degeneration distal to the injury^5^. Axonal regeneration occurs at variable rates (0.5–9.0 mm/day), depending on the type and severity of the injury, the condition of the target tissue, and the distance between the injury site and the end organ^6,7^. Maturation processes (remyelination, axonal enlargement, and establishment of connections with the end organ) often occur slowly before signs of functional recovery can be detected^5,7^. This underscores the need for non-invasive biomarkers capable of monitoring nerve regeneration throughout the recovery process to guide timely interventions.

Diffusion tensor imaging (DTI) has emerged as a non-invasive tool for assessing peripheral nerve injury and monitoring postoperative nerve recovery. DTI is a magnetic resonance imaging (MRI) based approach that probes information on tissue microstructure by measuring the effect of tissue barriers on the apparent diffusion coefficient (ADC) of water molecules within tissues^8–11^. In elongated cells such as nerve fibers, water diffusion is direction-dependent, with the ADC lower perpendicular to the fibers than along them. This anisotropic diffusion can be quantified using fractional anisotropy (FA), which ranges from 0 to 1, with higher values indicating healthy, well-organized nerve fibers and lower values indicating disrupted or injured nerve tissue^12^. Prior studies have suggested that FA relates to axon density^13^ and can reliably differentiate healthy and injured nerves^10,14^. Additionally, we have shown that DTI can distinguish varying degrees of partial nerve injury and detect failed nerve repairs^12,15^.

Despite its promise, the interpretation of DTI in peripheral nerve repair remains challenging. In this study, we analyze the longitudinal evolution of FA profiles along the distal segment of the sciatic nerve to characterize nerve health and recovery. These profiles are shaped by complex spatial and temporal interactions among multiple regenerative mechanisms, making them difficult to interpret reliably. Prior studies have primarily focused on isolated distal segments, limiting their ability to capture the full extent and heterogeneity of nerve regeneration.

To overcome this limitation, we apply a Gompertz function (a sigmoid model) to automate the characterization of FA profiles along the length of the nerve. FA profiles would be horizontally flat in healthy nerves with overall high FA. Meanwhile, FA profiles of injured nerves will look like a sigmoid function: 1) high FA describing the healthy nerve section above the injury site; 2) low FA representing the region undergoing nerve regeneration below the injury site and 3) region connecting the healthy (high FA) section of the nerve with the section undergoing nerve regeneration (low FA) section that will correspond to the injury site. This model has been successfully applied in growth models^16–18^ in multiple areas, including wound healing^19^, spinal cord injury recovery^20^, and behavioral recovery after traumatic peripheral nerve trauma^21^. We hypothesize that the Gompertz function can similarly enhance our ability to quantify and interpret changes in FA associated with nerve degeneration and regeneration, ultimately improving our ability to monitor recovery following surgical repair.

More specifically, this study presents a case series evaluating the utility of DTI and a mathematical model to monitor axonal regeneration after surgical repair of three injured and five healthy peripheral nerves, alongside sensory and motor assessments^3,14,21–23^. By applying the Gompertz function to FA profiles, we provide a preliminary evaluation of an automated, non-invasive approach for characterizing recovery and identifying patients who may benefit from additional surgical intervention.

## Results:

Clinical sensory and motor (S&M) assessments of repaired nerves were conducted approximately at 1- and 11/12- months post-surgery, as shown in Table 1^1,24^. For the median injured nerve of the right nerve in the first patient identified as MR1, the S&M score was S2/M2 one-month post-surgery and improved to S2/M3 by eleven months. At one-month post-surgery, evaluations of sensory and motor indices were established at S1/M1 in the ulnar nerve of the left arm of second patient identified as UL2 and in the median nerve of the right arm of same patients identified as MR2. Subsequently, at 12 months, the sensory and motor indices of UL2 were established at S3/M3, while the sensory and motor indices of MR2 were established at S2/M3.

**Figure 1.**
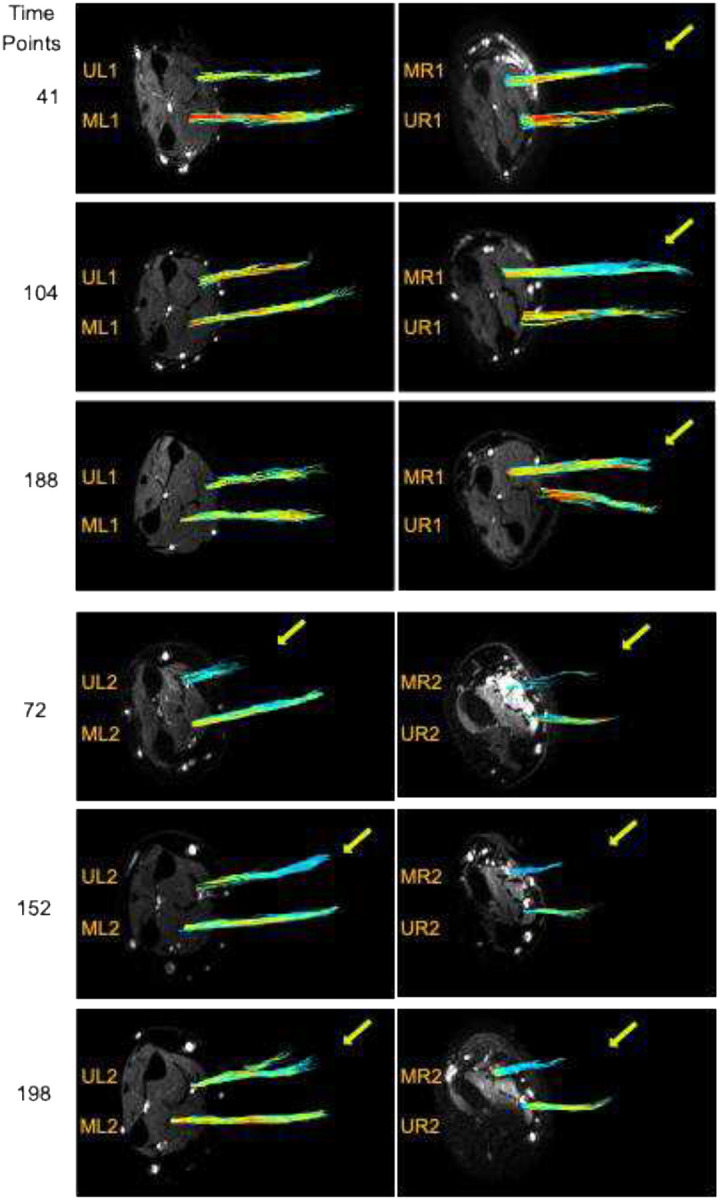
A) Tractography streamlines for nerves UR1, UL1, MR1, ML1, UR2, UL2, MR2 and ML2 for three different timepoints. Tracks are color-coded by FA, with blue indicative of low FA and red indicative of high FA. Streamline discontinuity occurs when FA are lower than 0.2. Yellow arrows indicate the injured nerve across all three timepoints. Images of the same nerve at different timepoints are shown stacked with timepoint indicated on the left side.

Figure 1 shows tractography results for all nerves. In the left arm of the first patient, ulnar and median nerves (UL1, ML1) are both healthy, displaying predominantly red and yellow color-coded streamlines, indicative of high FA. The healthy UR1 shown also displays yellow and red streamlines, again indicating high FA. In contrast, the injured median nerve in the right arm (MR1), indicated with a top right, yellow arrow, exhibits blue and light streamlines, indicative of lower FA and disrupted fiber integrity. The injured Ulnar nerve of the left arm in patient #2 (UL2) exhibits disrupted tracks at early time points, gradually transitioning into continuous dark blue color-coded tracks at later time intervals. Meanwhile, tracks of the healthy Median nerve (ML2) are blue-green and yellow at early time points, evolving into a yellow-green and later yellow-red color-coded tracks suggesting initially low FA transitioning to higher FA with time. The patient’s right arm exhibits an injured Median nerve (MR2) indicated with a yellow arrow. At the initial time-points, there is an apparent discontinuity and scarcity of tracks color-coded in dark blue. It can be seen that over time MR2 growths in terms of length, density, and thickness; however, tracks remain dark blue even at later timepoints that suggests low FA. At early time-points, DTI tracks of the healthy Ulnar nerve’s (UR2) without arrow are color-coded in blue-green near the injury and yellow far from the injury at early time points. Over time, the colors transitioned to yellow-green at mid-time points and yellow-red-coded tracks, indicating a transition from mid-range FA to high FA. Post-injury, at early time-points the included axial b0 MRI clearly depicts extensive inflammation in the right arm, manifested as a larger white bright area. Furthermore, it is observed that the left arm images are affected by field distortion, visible as a darker and black region originating from a metal plate located in the patient’s elbow due to previous injuries

**Figure 2.**
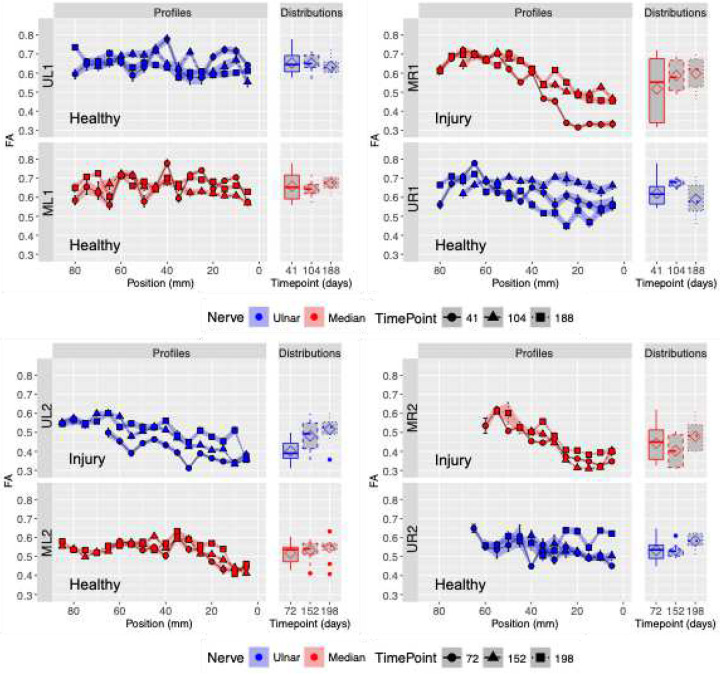
Slice-wise FA profiles for nerves UR1, UL1, MR1, ML1, UR2, UL2, MR2 and ML2 at three different timepoints. Twenty-eight nerves profiles organized by timepoints, and nerve and arm are shown here. Each set of nerves is split into two sections, with the FA profiles on the right and the corresponding FA distributions on the left. Ulnar nerves UL1, UR1, UL2 and UR2 in blue and median nerves ML1, MR1, ML2 and MR2 in red are presented in similar arrangement as tractography images for clarity. The shape of the data points in the FA profiles represent the first (circle), second (triangle), and third (square) scans. In addition, FA distributions are shown as boxplots with mean values indicated via a diamond.

Figure 2 presents the slice-wise mean FA grouped by nerve and timepoint. Ulnar (blue) and median (red) nerves are presented matching the same order used for Figure 1. Boxplots display the FA distributions, with mean values marked by diamonds. Aligning FA means of each slice across the nerve length forms the FA profile for each nerve.

It can be seen how healthy nerves, UL1, ML1, UR1 ML2 and UR2 show a relatively flat FA profile across all time points, with narrow boxplot distributions and consistent means. Specially in the case when both nerves of the same arm are healthy line in the case of UL1 and ML1. In contrast, injured nerve profiles exhibit a sigmoidal shape characterized by:

1. a high-FA upper asymptote on the left side of the profile (proximal region),
2. a low-FA lower asymptote on the right side (distal region, near the wrist),
3. increased variability and upward shifts in mean FA over time.

These features (along with the broader distributions shown in the boxplots) are identified in nerves MR1, UL2 and MR2. All other nerves showed non-sigmoidal shape and high-FA profiles consistent with healthy nerves.

To quantify these profiles, the Gompertz function was fitted to each FA curve as seen in Supplemental Figure 1. Parameter values derived from the fits each nerve and each time point, are provided in Table 2. Parameter sets from the injured nerves MR1, UL2 and MR2 are highlighted with a grey background. In the case of the first patient, FA_0_ of nerves UL1, ML1, MR1 and UR, remained constant, independently of the arm and time. In the case of the second patient, FA_0_ of nerves ML2 and UR2 remained constant and also for most of the FA_0_ of nerves MR2 and UL2. However, a slight reduction of the FA_0_ in the first timepoint of nerve UL2 can be noticed that is due to a short profile with lacking enough values at the proximal region. Similarly, a slight reduction of FA_0_ can also be noticed at the second timepoint of nerve MR2 whose profile also suffered of a shorter profile with less points at the proximal region. It can be noticed that FA_0_ also has two main values, 0.68 and 0.56 characteristics of each patient. In general, ΔFA indicated that FA at the distal region were lower than FA at the proximal region for all nerves. However, ΔFA in our first patient were ≤0.18 in healthy nerves, and mostly ≥0.18 in the injured nerves across all time points. Meanwhile, ΔFA in our second patient were lower than 0.11 in healthy nerves, whereas in injured nerves, ΔFA are mostly larger than 0.10. FA_Target_ in healthy nerves did not show a clear pattern, except that most were ≥0.5 in our first patient and ≥0.45 in our second patient. In contrast, injured nerves had FA_Target_ <0.5 and <0.44 respectively. X_I_ does not show any pattern over time and was characterized by larger error values in healthy nerves than injured nerves. Finally in the case of our first patient, FA_I_ exhibited values >0.61 in the healthy nerves, and <0.61 in the injured nerves while in the second patient FA_I_ exhibited values >0.519 in healthy nerves and < 0.517 in injured nerves. In the case of FA_I_, neither healthy nor injured nerves exhibited an evolutionary pattern.

**Figure 3.**
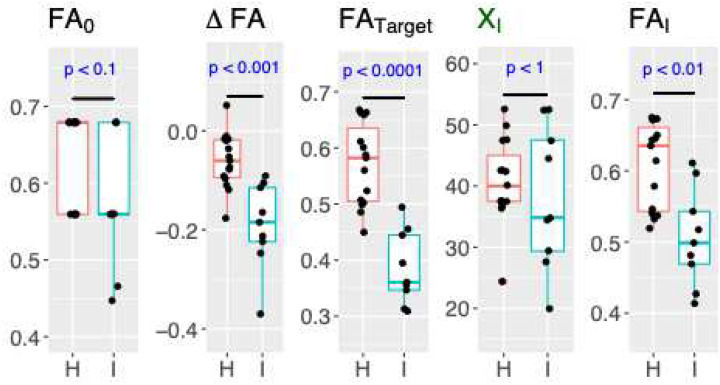
Boxplot representation of variables ΔFA, FA_Target_ and FA_I,_ X_I_ and FA_0_ segregated by healthy nerves (H; red) or injured nerves (I; Blue). P value calculated using a two-sided non-paired Wilcox test (blue text)

**Figure 4.**
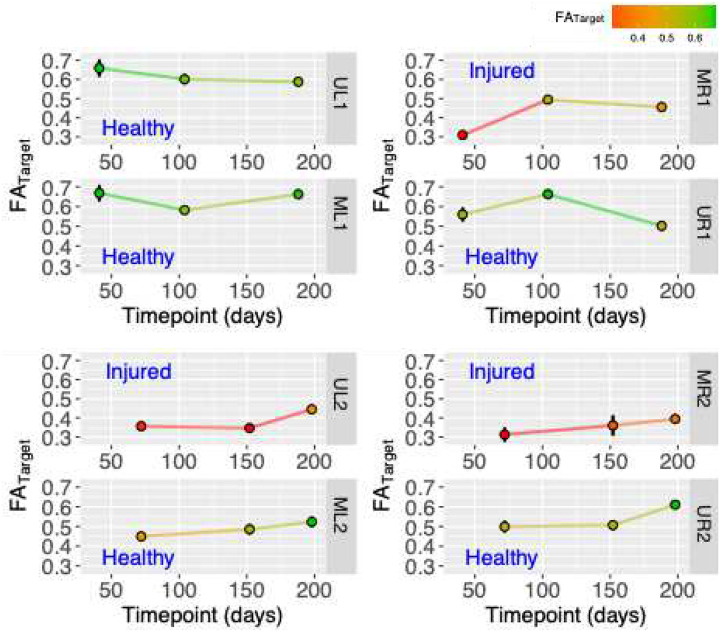
Longitudinal evolution of FA_Target_ values of each nerve with color-coded lines and dots based on the FA value. Nerve arrangement follows tractography and FA profiles that in previous figures.

Figure 3 combines the measurements from all nerves (Table 2), independently of the timepoint and identifies significant differences for the variables ΔFA (p < 0.001), FA_Target_ (p <0.0001) and FA_I_ (p<0.01) when injured (I) and healthy (H) nerves are compared. Based in these p-values, we picked FA_Target_ to study and quantify nerve recovery of injured nerves. The longitudinal evolution of each nerve’s FA_Target_ is shown in Figure 4. FA_Target_ values of the healthy arms UL1 and ML1 are depicted as bright green with high FA at all timepoints presenting little variability over timepoints. Healthy nerves located in arms that suffered an injury (UR1, ML2 and UR2) are initially characterized by lower FA_Target_ values (orange to orange-green) and over time mostly increase to high FA (bright green). Finally, all three injured nerves (MR1, UL2, and MR2) start initially with low FA (red) that will increase over time to higher FA (orange). In Figure 4 healthy nerves and injured nerves, can be easily identified and in addition nerve recovery, can be quantified.

**Figure 5.**
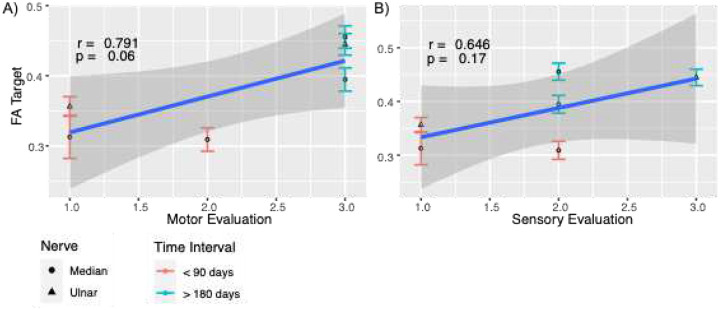
Correlation plots between A) FA_Target_ and Motor Evaluation and between B) FA_Target_ and Sensory Evaluation of injured nerves. Points are color-coded by the time interval when data was measured and shaped by the type of nerve (Median or Ulnar). Blue line represents an estimate of the best lineal fit with confidence band (dark grey). Correlations and p-values are included in top left section of each graph.

Figure 5 shows the correlation plots between FA_Target_ of injured nerves (MR1, UL2 and MR2) and the Sensory and Motor assessments. These plots suggest a strong correlation between FA_Target_ and Motor Assessment (r = 0.791) and a strong but more moderate correlation between FA_Target_ and Sensory Assessment (r = 0.646). In both cases, the low sample size is reflected in the form of p-values larger than 0.05. Furthermore, no significant differences between the Ulnar nerve (triangle shaped points) and the Median nerve (circular shaped points) could be detected.

## Discussion:

To evaluate the clinical applicability of FA in analyzing the progression of nerve regeneration, we performed a total of twenty-four MRI scans obtained from eight nerves that were scanned at three different timepoints. All these scans were obtained from two recruited patients who underwent nerve repair surgery in their forearms due to TPNI. Patient #1 sustained an injury to the median nerve in the right arm that resulted in three healthy nerves and one injured nerve, while patient #2 experienced injuries to both the median nerve in the right arm and the ulnar nerve in the left arm. Together, recovery was longitudinally assessed for three separate nerve injuries with surgical repair scenarios and five healthy nerves. In this case-control study we explored the potential of non-invasive DTI measurements as a tool to: 1) differentiate between healthy and injured nerves; 2) quantify the deficits post-TPNI relative to their normal state; 3) monitor and assess nerve regeneration over time. To accomplish this, we analyzed tractography images, FA distribution per nerve and timepoint and parametrized the FA profiles along each nerve segment near the injury and repair site. By comparing the measurements of each variable segmented by healthy and injured nerves, we estimated the sensitivity of each variable to nerve health. FA_Target_ was identified as the most suitable variable to analyze nerve recovery. Additionally, correlations between FA_Target_ and clinical sensory assessments and FA_Target_ and motor assessments supported FA_Target_ as a potential biomarker to assess the evolution of nerve recovery.

Clinical assessments evaluated sensory and motor function at one and eleven months after nerve repair. Table 1 shows that median nerve MR1 from patient #1 improved from S2/M2 to S2/M3. Table 1 also shows median nerve MR2 from patient #2 improved from S1/M1 to S2/M3, while the ulnar nerve UL2 improved from S1/M1 to S3/M3. These results indicate similar end motor recovery (M3) across nerves; however, sensory outcomes differed, with both median nerves ending at S2 while the ulnar nerve reached S3.

Tractography analysis (Figures 1) revealed extensive and densely packed fiber tracts in both patients’ healthy nerves UL1, ML1, UR1, Ml2 and UR2, predominantly colored yellow and red. In the injured nerve MR1, tractography at early timepoints indicated blue tracts at the nerve’s distal region, followed by an increase in yellow and red tracts in the final timepoint, suggesting ongoing recovery over time. The injured nerves UL2 and MR2 displayed track discontinuity at the first timepoint, suggesting severe nerve damage. Approximately 90 days later, there was an increase in the number and length of tracks in both nerves, displaying a bright blue color. However, there was still some tract discontinuity in MR2, suggesting a stronger recovery of UL2 compared MR2. At the final time points, UL2 exhibited a high density of fibers with blue and yellow tracks, suggesting an ongoing nerve recovery. Meanwhile, MR2 showed higher fiber density but exhibited low growth and retained its blue color, suggesting lower growth and recovery. Of note, field inhomogeneity from the metal plates in patient #2’s right arm produced dark MRI signal voids, likely contributing to the shorter and less dense tracts observed in nerves MR2 and UR2.

Figures 2 present FA profiles and boxplots of UL1, ML1, UR1, ML2 and UR2 healthy nerves from both patients, revealing consistent FA across all positions. This low variability results in predominantly horizontal profiles and narrow boxplots, consistent with a relatively uniform distribution of values. This suggests that the intrinsic structure of the nerves remains consistent along the arm. Furthermore, FA profiles and boxplots of healthy nerves exhibit minimal temporal changes, indicating stability over all time points. In contrast, the FA profiles in the three injured nerves MR1, UL2 and MR2 exhibit the following characteristics: 1) a horizontal asymptote characterized by high FA and situated far from the wrist (proximal region); 2) a progressive decline in FA as proximity to wrist increases (injury region); and 3) a second horizontal asymptote characterized by low FA located close to the wrist (distal region). The evolution of the profiles’ shapes over time serves as an indication of the longitudinal nerve recovery process.

The evolution of nerve MR1’s FA profiles over time reflects underlying longitudinal nerve recovery. In patient #1, the profiles became smoother across sessions, driven primarily by an increase in FA in the distal region between the first and second time points, with little additional change thereafter. In patient #2, both injured nerves UL2 and MR2 show progressive increases in distal FA across all time points, suggesting ongoing recovery. Although the boxplots of injured nerves also detect these longitudinal changes—visible as upward shifts in median FA—they show wide interquartile ranges. This variability reduces sensitivity to small changes in FA and makes subtle recovery-related trends harder to detect.

Parametrization of each FA profile was performed using the Gompertz function, providing quantitative values for the proximal, injury and distal sections. This process produces quantitative measurements (Table 2) characterized by: 1) reduced sensitivity to slice-level noise and 2) increased segmentation accuracy of FA profiles that are independent of user bias. Further analysis of all the calculated variables is shown in Figure 3. Here, the derived variables ΔFA, FA_Target_ and FA_I_ have the potential to identify healthy nerves from injured nerves and of them FA_Target_ is the variable that best discriminates between healthy and injured nerves (p<0.0001). This suggests that FA_Target_ has the potential to analyze the evolution of peripheral nerve post nerve repair injury and better correlate with clinical evaluation results. In contrast, FA_0_ corresponding to the proximal values used to normalize the profiles and X_I_ that describes the location of the value FA_I_ do not discriminate between injured and healthy nerves. It is worth mentioning that FA_0_ = 0.68 ± 0.03 in patient #1, and FA_0_ = 0.56 ± 0.02 in patient #2 were different. These differences seem to be attributed to inflammation and edema, as patient #1’s left arm was injury-free while patient #2 suffered injuries in both arms^28^.

Longitudinal evolution of nerve recovery for each nerve is shown in Figure 4. Here, we focused on FA_Target_ since it exhibited the most significant differences between injured and healthy nerves. Median and ulnar Nerves in Patient #1’s left arm (ML1 and UL1) are characterized by high FA_Target_ values at all timepoints (color-coded as bright green), presenting little variability over time. These values correspond to the only arm in the study where both ulnar and median nerves are free from injury and inflammation / edema. In contrast, MR1 of patient #1, is characterized by low FA (red) at the earliest timepoint followed by an increase to higher FA (orange or orange-green) over time.

These results indicate an injured median nerve that quickly recovers within the first 100 days, plateauing after that timepoint. Meanwhile, Patient #2’s ML2 and UR2, are initially characterized by mid-range FA_Target_ values (orange to orange-green) that increase over time to higher FA (bright green). This FA_Target_ evolution corresponds to two healthy nerves located in injured arms, suggesting that an initial decay in FA reflects inflammation in otherwise healthy nerves, with subsequent increases in FA corresponding to the resolution of inflammation. Additionally, UL2 and MR2 nerves are injured nerves. In both cases, the nerves initially exhibit low FA (red), which increase over time to moderate FA (orange). Further analysis of Figure 4 indicates that recovery patterns differ across patients and nerves. Patient #1 demonstrates greater improvement within approximately 170 days, whereas patient #2 exhibits a more gradual or delayed recovery. These differences may reflect injury type, age, overall health, or overuse of the dominant side. DTI-derived FA measurements, combined with modeling, may help support earlier identification of cases that could require reoperation.

Early time points (red) in Figure 4 exhibited lower FA and lower sensory/motor scores, while later time points (blue) exhibited higher FA and higher sensory/motor scores. This suggests potential time-dependent relationships between behavioral and MRI metrics. Correlations between motor assessments and FA_Target_ parameters (Figure 5 A), and between sensory assessments and FA_Target_ parameters (Figure 5 B), support the potential utility of FA_Target_ as a biomarker, with a strong correlation observed for motor scores (r = 0.79) and a more moderate correlation for sensory scores (r = 0.65). However, the small sample size may have resulted in limited statistical power and contributed to the non-significant correlations observed herein. The early (<90 days) measurement of patient #1’s median nerve may represent an outlier, and a larger sample will be needed to mitigate the influence of such points. Normalization resulted in a stronger correlation between FA_Target_ and MRC clinical scores (Figure 4), favoring FA_Target_ over ΔFA as a candidate biomarker of recovery due to its smaller relative uncertainty.

This study is limited to five healthy nerves and three TPNI. DTI was analyzed on an individual nerve level across three time points whereas clinical assessments were available only at two. FA_Target_ reduced noise but did not eliminate FA variability, potentially influenced by patient discomfort and motion. The sensory and motor recovery outcomes were obtained indirectly through a retrospective chart review, which significantly limits the robustness of our clinical data. Future studies that include standardized sensory and motor examinations for MRC grading at each DTI scan time point will provide stronger clinical data and allow for more reliable validation of the DTI findings. Future studies will also require larger cohorts, more frequent imaging time points, and standardized clinical assessments.

In this study, FA from DTI matched clinical assessments and levels of nerve recovery. Across all subjects, FA_Target_ were: i) significantly reduced in TPNI relative to healthy control nerves, ii) FA of healthy nerves are higher when no injury was present in that arm, iii) nerves showed FA a consistent with changes in S&M measurements, suggesting the capacity to accurately determine degeneration and potential levels during the regeneration process. Tractography images clearly differentiated between healthy nerves and injured nerves but are largely qualitative. In contrast, nerve FA profiles quantitatively differentiated between healthy and injured nerves based, potentially providing a biomarker of time-dependent nerve recovery. However, analyzing these profiles using conventional methods resulted in significant user-dependent variability. Here, we deployed a reliable Gompertz-based model to automatically and consistently extract the proximal, injury, and distal regions, reducing variability and improving the sensitivity of the measurements. Our results indicate that the derived parameter FA_Target_ can accurately categorize nerve recovery over time, supporting its ability to identify early recovery or failure and determine whether a second intervention may be needed.

## Methods:

### **Ethics approval**.

This study was conducted in accordance with the guidelines of the Institutional Review Board at Vanderbilt University Medical Center (IRB #231846) and the Health Insurance Portability and Accountability Act (HIPAA) to ensure the ethical handling of patient data. The experimental protocols were reviewed and approved by the IRB committee, and all methods were performed in accordance with its guidelines and regulations, including the Declaration of Helsinki. Informed consent was obtained from all participants and/or legal guardians prior to inclusion in the study. All patient data was de-identified before analysis to maintain confidentiality. Data collection, storage, and processing adhered to institutional security protocols, including encryption and restricted access to authorized personnel. Inclusion criteria corresponded to patients aged 18 to 70 years with Sunderland IV (axonotmesis) or V (neurotmesis) upper extremity nerve injuries who underwent surgical repair. Our exclusion criteria contained patients with pre-existing peripheral neuropathy, contraindications to MRI scans such as claustrophobia or presence of metal objects, inability to complete follow-up visits, and or pregnant or breastfeeding

### Experimental design.

Twenty-four MRI scans from a total of eight nerves were obtained resulting in five healthy nerves and three nerves suffering from traumatic peripheral nerve injury. Patients with severe peripheral nerve injuries were recruited for this study, as seen in Table 1 and included a 33-year-old female, suffered from a complex forearm laceration in the right arm. The laceration was located at the wrist, involving tendon injury and caused median nerve transection. This patient was treated with a median nerve group fascicular repair. Additionally, a separate 39-year-old female was included that suffered from bilateral nerve injuries including a Median nerve injury located in the right forearm. Additionally, this patient also suffered damage to the ulnar nerve in the left arm. Both nerve injuries were treated via nerve repair surgery including group fascicular repair and epineural repair. Clinical sensory exams, motor function exams, and DTI scans were performed between 1- and 12-months after nerve repair surgery^1,24^.

### MRI protocols.

MRI data was acquired in each subject using a Philips 3.0-T Ingenia CX scanner with a small extremity 8-channel coil. Subjects were scanned in prone or lateral decubitus position, with one arm extended over the head and centered within the bore of the scanner. Both arms were scanned within the same session, with a short break taken between arms to reposition the subject. MRI scans were obtained at 41, 104, 188 days and at 72, 152, 198 days postoperatively for patients #1 and #2.

Structural imaging (Figure 5) included both a T1-weighted turbo spin echo (T1W TSE) sequence and the following parameters (field-of-view = 100×121×100 mm^3^, resolution = 0.347×0.347×4.5 mm^3^, TE/TR = 13/682 ms, averages = 2, scan time = 1min 3 sec) and T2-weighted TSE sequence (field-of-view = 100×121×100 mm^3^, resolution = 0.26×0.26×4.5 mm^3^, TE/TR = 75/4083 ms, averages = 2, scan time = 1 min 46 sec). For DTI, data were collected with a multi-slice diffusion-weighted echo-planar imaging (EPI) sequence with the following parameters: field-of-view = 100×100×80 mm^3^, resolution = 1×1×5 mm^3^, TE/TR = 75/3000 ms, b-value = 1000 s/mm^2^, 20 diffusion-encoding directions, 1 b = 0 s/mm^2^ image, and scan time = 10 min and 33 s.

### DTI analysis.

Each MRI session (Supplemental Figure 1A) produced T1w and T2w anatomical images (Supplemental Figure 1 B), that were used for guidance in the DTI analysis, in addition to FA maps (Supplemental Figure 1 C). Regions of interest (ROI) were drawn manually in each axial cross-section of the FA maps to calculate the mean slice-wise FA. In general, the proximal region was characterized by high FA (Supplemental Figure 1 D). Meanwhile, the area corresponding to the injury was characterized by a sharp decline in FA (Supplemental Figure 1 D). Finally, the region distal to the injury was characterized by lower FA, likely related to Wallerian degeneration and axonal regeneration postoperatively (Supplemental Figure 1 D).

Diffusion tensors were estimated via weighted linear least-squares regression on a voxel-wise basis using ExploreDTI Toolbox in MATLAB^25,26^. Tractography was performed using two seeds, both located in the proximal region, in the ExploreDTI Toolbox. The first seed was situated at the second-to-last slice, and the second seed was located at the fourth-to-last slice. The minimal value for FA tracking was 0.25, with a maximal angle of 30 °, a step size of 0.3 mm, and minimal/maximal fiber lengths of 20/5000 mm, respectively. Tractography in healthy nerves is characterized by long, densely pack fiber tracks with mostly yellow and red color-coded tracks (mid/high FA). In contrast, tractography of injured nerves is characterized mostly by: 1) low density fiber tracks specially at early timepoints; 2) a discontinuation of the fiber tracks when FA is below the FA boundaries during the reconstruction; 3) Blue color-coded fiber-tracks (low FA).

### Clinical Assessment.

A retrospective chart review of nerve-specific sensory and motor clinical exams reported during clinical visits was conducted to obtain data for the Medical Research Council (MRC) sensory and motor grading, with sensory grades S0–S4 representing absent to normal sensation, and motor grades M0–M5 representing muscle contraction to normal strength)^27^. Sensory recovery scores are stablished as: S0) absence of sensibility in the autonomous nerve; S1) recovery of tactile sensibility and deep cutaneous pain; S2) recovery of superficial cutaneous pain and tactile sensibility; S3 return of pain and tactile sensibility without over response and S4) complete recovery. Meanwhile, muscle strength scores are scaled as: M0) no contraction, M1) flicker or trace of contraction; M2) Full range of active movement without gravity; M3) active movement against gravity; M4) active movement against gravity and resistance and M5) Normal power^27^.

### Statistical analysis

All statistical analyses were performed using R (version 4.3.1). For each MRI slice, ExploreDTI was used to compute the mean FA and standard error across the nerve cross-section. The slice-wise FA were ordered anatomically to generate FA profiles spanning the proximal (healthy), injury, and distal regions of each nerve. FA distributions of the full profile were visualized using box-and-whisker plots, with mean values shown as diamonds.

For each nerve and timepoint, FA profiles were then fit to a Gompertz function (Equation 1, as shown in the Figure 2E) using nonlinear regression (NLS library, stats version 3.6.1)

(1) $$FA\left(x\right)={FA}_{0}+\Delta FA\cdot {e^{-e}}^{\left(-b\cdot (x-x_{I})\right)}.$$

Each fit produced a sigmoid curve with parameters describing key biological features of nerve injury and regeneration. The upper asymptote (FA_0_) represents the FA of the proximal, healthy portion of the nerve. The lower asymptote (FA_Target_) represents FA at the distal region after injury. ΔFA quantifies the difference between these two regions. The inflection point (x_i_) indicates the slice location with the steepest FA change, and FA at this location is FA_i_ = FA_0_ + ΔFA/e. Parameter estimates are reported as mean ± standard error.

Because FA measurements can vary across sessions due to experimental factors, a baseline correction was applied. For each subject and time point, FA_0_ was compared with the subject-specific mean FA_0_ across all time points. Each FA profile was then normalized using this difference, producing FA_Target_ as a standardized measure of nerve health and recovery. Finally, Pearson correlations were used to evaluate the relationship between FA_Target_ and sensory and motor behavioral scores across time intervals. Data from both subjects were combined for this analysis and resulting correlation coefficients (r) and p-values were used to assess the strength of association between DTI metrics and functional outcomes.

## Supplementary Material

Supplementary Files

This is a list of supplementary files associated with this preprint. Click to download.

- 20260331Supplementary.docx

## Figures and Tables

**Figure 6.**
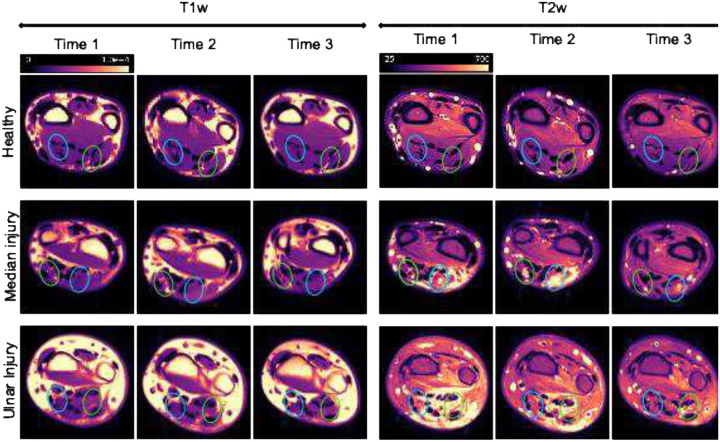
Representative Images: T1 weighted and T2 weighted show Healthy, Median and Ulnar traumatic peripheral nerve injuries (TPMI) cohorts taken at Time-1, −2 and −3. Green circles indicate the Ulnar nerve. Blue circles indicate the Median nerve.

**Table 1.** Demographic information for all subjects. Clinical assessments of repaired nerves S&M of patients are shown at the specified postoperative time points (months). Data were extracted from clinical notes. N/A indicate healthy nerve indicating not injury or treatment. Age is expressed in years and sex is indicated as Female (F) and Male (M)

Patient			Demographics					Clinical Scores	
	Age (Sex)	Arm	Nerve	ID	Injury Type	Treatment	Time point (Months)	Sensory	Motor
#1	33(F)	Left	Ulnar	UL1	N/A	N/A			
			Median	ML1	N/A	N/A			
		Right	Ulnar	UR1	N/A	N/A			
			Median	MR1	Nerve laceration	Nerve Repair via Suture	1	S2	M2
							11	S2	M3

#2	39(F)	Left	Ulnar	UL2	Nerve laceration	Nerve Repair via Suture	1	S1	M1
							12	S3	M3
			Median	ML2	N/A	N/A			
		Right	Ulnar	UR2	N/A	N/A			
			Median	MR2	Nerve laceration	Nerve Repair via Suture	1	12	M1
							12	S2	M3

**Table 2.** Parameters obtained from fitting the Gompertz function to the FA profiles for all nerves. The injured median nerve in highlighted by the gray box.

Nerve	Timepoint	FA_0_	ΔFA	FA_Target_	X_i_	FA_i_
UL1	41	0.68±0.04	0.02±0.08	0.66±0.04	38±88	0.672±0.004
	108	0.68±0.02	0.08±0.07	0.60±0.02	43±15	0.650±0.02
	188	0.68±0.01	0.09±0.03	0.59±0.014	38±7	0.6454±0.0005
ML1	41	0.68±0.03	0.01±0.01	0.67±0.03	48±205	0.675±0.007
	108	0.68±0.01	0.10±0.05	0.58±0.012	43±9	0.643±0.0012
	188	0.68±0.02	0.02±0.06	0.66±0.02	48±65	0.673±0.002
MR1	41	0.68±0.02	0.37±0.04	0.31±0.02	44±2	0.5430±0.0008
	108	0.68±0.01	0.18±0.02	0.49±0.010	35±2	0.6111±0.0002
	188	0.68±0.02	0.22±0.05	0.46±0.02	48±4	0.5967±0.0012
UR1	41	0.68±0.03	0.12±0.06	0.56±0.03	38±11	0.635±0.002
	108	0.68±0.01	0.02±0.04	0.66±0.013	43±43	0.6732±0.0009
	188	0.68±0.02	0.18±0.03	0.50±0.02	38±4	0.6141±0.0007
UL2	72	0.45±0.01	0.09±0.02	0.36±0.014	3 ±15	0.4136±0.0004
	152	0.56±0.01	0.21±0.04	0.35±0.013	53±4	0.4812±0.0008
	198	0.56±0.02	0.11±0.05	0.44±0.02	53±8	0.517±0.0011
ML2	72	0.56±0.01	0.11±0.03	0.45±0.01	40±4	0.5191±0.0006
	152	0.56±0.02	0.07±0.06	0.49±0.02	50±16	0.532±0.002
	198	0.56±0.02	0.04±0.06	0.52±0.02	53±36	0.546±0.002
MR2	72	0.56±0.03	0.25±0.04	0.31±0.03	28±5	0.469±0.003
	152	0.47±0.05	0.10±0.02	0.36±0.05	20±2	0.427±0.004
	198	0.56±0.02	0.16±0.05	0.39±0.02	29±2	0.4989±0.0005
UR2	72	0.56±0.02	0.06±0.06	0.50±0.02	36±14	0.5373±0.0010
	152	0.56±0.02	0.05±0.04	0.51±0.02	24±27	0.5403±0.0005
	198	0.56±0.02	0.05±0.03	0.61±0.02	37±18	0.5785±0.0005

## Data Availability

The data related to the current study will be made available from the corresponding author upon reasonable request.
